# Associations between animal and herd management factors, serological response to three respiratory pathogens and pluck lesions in finisher pigs on a farrow-to-finish farm

**DOI:** 10.1186/s40813-020-00173-z

**Published:** 2020-12-08

**Authors:** Rose Mary Fitzgerald, Helen O’Shea, Edgar García Manzanilla, John Moriarty, Hugh McGlynn, Julia Adriana Calderón Díaz

**Affiliations:** 1 Bio-Explore, Department of Biological Sciences, Cork Institute of Technology, Rossa Avenue, Bishopstown, Cork, T12 P928 Ireland; 2 Pig Development Department, Teagasc Animal and Grassland Research and Innovation Centre, Moorepark, Fermoy, Co. Cork P61 C996 Ireland; 3 School of Veterinary Medicine, University College Dublin, Belfield, Dublin 4, D04 V1W8 Ireland; 4 Central Veterinary Research Laboratory, Department of Agriculture, Food and the Marine Laboratories, Backweston, Celbridge, Co. Kildare W23 X3PH Ireland

**Keywords:** *Actinobacillus pleuropneumonia*, All-in/all-out, Enzootic pneumonia, Lung lesions, *Mycoplasma hyopneumoniae*, Pleurisy, Serology, Sow parity, Swine influenza virus

## Abstract

**Background:**

Serological screening is a common method to monitor antibody response to pathogen exposure, but results could vary due to several factors. This study aimed to quantify animal and management related factors associated with variation in antibody levels in finisher pigs at slaughter, in an Irish farrow-to-finish farm endemically infected with *Actinobacillus pleuropneumonia* (App), *Mycoplasma hyopneumoniae* (Mhyo) and swine influenza virus (SIV). A second objective was to estimate differences in antibody levels in pigs presenting pluck lesions. This was an observational study whereby pigs were managed as per routine farm practice. Data on sow parity, number of born alive (NBA) pigs per litter, cross-fostering status, birth and weaning body weight were recorded from 1016 pigs born from one farrowing batch. At slaughter, blood samples were collected for serological analysis and pigs were inspected for presence of enzootic pneumonia (EP)-like lesions, pleurisy, pericarditis and heart condemnations. Pigs were retrospectively classified into three production flows, depending on time spent in each production stage: flow 1 (F1; pigs followed the normal production flow); flow 2 (F2; pigs which were delayed by 1 week from advancing forward); and flow 3 (F3; pigs delayed by > 1 week from advancing forward). A nested case-control design was applied by matching pigs from each flow by sow parity, birth weight and NBA.

**Results:**

Pigs born from primiparous sows had higher antibody levels for App than those born to parity ≥5 sows (*P* < 0.05) and there was no association between any of the pathogens investigated and other early life indicators (*P* > 0.05). Pigs in F1 had lower antibody levels for App but higher antibody levels for SIV than F2 and F3 pigs (*P* < 0.05). There was no association between pluck lesions and respiratory pathogens (*P* > 0.05), except for increased antibody levels for Mhyo when EP-like lesions were present (*P* = 0.006).

**Conclusion:**

Results indicate that offspring from primiparous sows develop higher antibody levels for App IV toxin when exposed to this disease and that enforcement of a strict all-in/all-out production system would reduce on-farm disease circulation. A high percentage of pigs were affected with EP-like lesions which were associated with higher antibody levels for Mhyo.

## Background

Respiratory diseases are among the most significant infectious health issues within the pig production industry worldwide, facilitated by the current intensification structure of production [1]. *Actinobacillus pleuropneumoniae* (App), *Mycoplasma hyopneumoniae* (Mhyo) and swine influenza virus (SIV) are among the most important respiratory pathogens in pigs; they are agents involved in the porcine respiratory disease complex [2] and the occurrence of pneumonic and pleuritic lesions [2–4]. Respiratory pathogens are mainly transmitted between herds by introducing carrier pigs to naïve populations [5] or by inadequate biosecurity measures [6]. Once introduced into the herd, App and Mhyo become endemic in nearly all cases [5, 7] and SIV appears to persist in farrow-to-finish farms, where susceptible piglets are always available [8], with outbreaks mostly occurring during the fall and winter seasons when temperatures start to drop [8].

In endemically affected herds, respiratory pathogens infection is maintained by vertical transmission from infected sows to their offspring [9] and by lateral transmission post-weaning, when maternal immunity decreases and/or by mixing naïve pigs with pathogen carriers [10]. Vertical transmission is associated with the quantity of pathogens shed by the sow [11] and the level of antibodies transferred from sow to piglets in the colostrum [11, 12]. For instance, it is reported that gilts shed more microorganisms, and are more likely to transmit pathogens to their offspring [13] due to inferior quality colostrum [14] than older sows, suggesting that piglets born to gilts would acquire lower quality passive immunity [14]. Other factors could also affect the level immunity transferred from dam to offspring. Early-life indicators such as birth body weight and weaning body weight, litter size and cross-fostering status are associated with higher susceptibility to App and Mhyo and the presence of pluck lesions [2, 15].

Lateral transmission occurs by pig-to-pig contact between infected and susceptible animals. An effective way to reduce disease spread is the implementation of strict all-in/all-out (AIAO) management practices [16, 17]. In true AIAO systems, pigs are closely matched by age and move forward through the production stages in the same groups reducing disease transmission and improving animal performance [18]. However, implementation of strict AIAO practices is difficult in farrow-to-finish farms as the majority lack facilities exclusively dedicated to *pull outs* (i.e. slow-growing and/or sick pigs). Pigs are often regrouped at various times according to their body weight [2], in an effort to achieve uniformity in slaughter weight [16]. This results in creating, perhaps inadvertently, several “production flows” increasing the likelihood of disease transmission between pigs of different age groups [2] with mixed immune status [19], within and between flows and extending to subsequent farrowing batches.

Disease surveillance is key to effective disease management by identifying risk factors and monitoring the spread of disease to manage it effectively [20]. Serological tests such as enzyme-linked immunosorbent assay (ELISA) tests are usually used for disease surveillance because they are faster, simpler to perform and more cost effective than other methods [21]. ELISA test results are an indicator of seroconversion following vaccination or of field exposure to a pathogen although they are not necessarily a reflective measure of disease protection [22]. However App and Mhyo antibody seroprevalence are associated with severity of lung and pleural lesions [23]. Antibody levels could vary due to several factors including amount of antibodies transferred from sow to offspring for a specific pathogen [24] and humoral immune response [25].

Thus, the objective of this study was to quantify animal and herd management factors associated with variation in antibody levels in finisher pigs at slaughter, in a farrow-to-finish commercial farm with presence of endemic App, Mhyo and SIV. A secondary objective was to estimate the association between antibody levels of App, Mhyo and SIV with pluck lesions. We hypothesise that pigs originating from younger sows, light weight pigs at birth and/or weaning, pigs cross-fostered during lactation and pigs repeatedly delayed from advancing through the different production stages would show an increased antibody level to respiratory pathogens present in the farm. We also hypothesise that mean antibody levels would be higher for pigs presenting pluck lesions at slaughter.

## Methods

### Pig housing and management

This study was completed on a 1500 Large White × Landrace sow farrow-to-finish Irish commercial farm, with weekly farrowing batches of approximately 80 sows. The farm was seropositive for App, Mhyo and SIV but seronegative for porcine respiratory and reproductive syndrome virus. The farm vaccinated piglets against Mhyo between 10 to 12 days of age and at weaning (i.e. 28 days of age), while a blanket SIV vaccination was used for sows every 6 months with a trivalent vaccine. This commercial farm purported that it implemented an AIAO production system, with batches of pigs post-weaning, progressing to the first nursery stage (4 weeks), then advancing to the second nursery stage (4 weeks), moving to the grower stage (4 weeks) and finally to the finisher stage (8 weeks). This was an observational study, whereby pigs were managed as per routine farm practice and the weekly movement of animals was tracked. Calderón Díaz et al. [26] and Diana et al. [27] previously described information regarding animal management, associations between production flow and animal performance and health (including pluck lesions [26]) indicators at slaughter and between production flows and welfare indicators during the grow-finisher period [27].

In brief, a total of 1016 pigs, born within one weekly farrowing batch, were followed through the different production stages. All pigs were individually tagged at birth and information on sow parity, number of piglets born alive per litter (NBA), sex and cross-fostering status (i.e. cross-fostered or not cross-fostered) was recorded. All pigs were weaned at approximately 28 days of age. At weaning, entire litters were transferred to the first nursery stage and housed in groups of 55 pigs, (minimum of 0.30 m^2^ pen space per pig) comprised of 4 to 5 litters. During the second nursery and grower stage, pigs were split and regrouped according to size and/or body weight (BW) and housed in groups of 36 animals, with a minimum of 0.55 m^2^ pen space per pig. Upon transfer to the finisher period, pigs were housed in groups of 35 pigs, with a minimum of 0.65 m^2^ pen space per pig. Housing facilities were uniform (i.e. pens, floor surface and ventilation system) within each of the production stages. Nursery and growing facilities had an automatic temperature control system with ceiling fans, while finisher facilities had natural ventilation. In all stages, animals were housed on fully slatted floors; plastic floors for nursery and concrete floors for the grower and finisher stages. Pigs were provided with wet-feed ad libitum during nursery; [18.3% crude protein (CP) and 10.5 MJ/DE per kg of feed]; grower (18.1% CP and 10.0 MJ/DE per kg of feed) and finisher diets (16.9% CP and 9.9 MJ/DE per kg of feed). Pigs had ad libitum access to water via a nipple drinker for each 10–15 pigs.

Mortality was recorded during the study. A total of 145 pigs died and 47 pigs were euthanised during the various phases of production representing 18.9% of all pigs in the study. Regarding mortality, 104 pigs died during lactation (54.2% out of 192 pigs), 24 pigs died during the nursery stages (12.5% out of 192 pigs), 3 pigs died during the grower stage (1.5% out of 192 pigs) and 14 pigs died during the finisher stage (7.3% out of 192 pigs). The remaining 47 pigs (24.5% out of 192 pigs) were selected for euthanasia due to the presence of abnormalities such as external lesions, hernias, tail loss, severe lameness, external abscesses and emaciation. Eight-hundred-and-twenty-four pigs reached slaughter age and they were slaughtered within 1 week, regardless of body weight at 24 weeks of age, for the purpose of the experiment. Pigs were retrospectively classified into three production flows, depending on the time spent in each production stage: flow 1 = pigs that advanced through the normal production stages ‘in a timely manner’; *n* = 620; flow 2 = pigs which were delayed by 1 week from advancing forward to the next production stage; *n* = 111; and flow 3 = pigs delayed by more than 1 week from advancing to the next production stage; *n* = 93.

### Blood sampling and serological analysis

At slaughter, individual blood samples were obtained at exsanguination using labelled red-stopper sterile BD Vacutainer® blood collection tubes (Becton, Dickinson U.K. Ltd., Berkshire, U.K.). All blood samples were individually labelled on collection, with corresponding sample delivery documents. Samples were transported to the Irish Department of Agriculture, Food and the Marine’s, Blood Testing Laboratory, Cork, for analysis. Blood samples were processed following clot formation, with serum aliquoted into individually labelled, anonymised cryovials (STARSTEDT®, Nümbrecht, Germany) and stored at -80 °C until required for analysis. All serum samples were analysed by ELISA using commercial pathogen-specific ELISA kits (IDEXX Europe B.V., Hoofddorp, The Netherlands) for the three respiratory pathogens of interest [App - ApxIV (Apx IV toxin is produced during an episode of infection which is common and specific to all serotypes) Ab Test (97.8% sensitivity, 100% specificity); Mhyo – HerdChek® *Mycoplasma hyopneumoniae* Antibody Test (89.4% sensitivity, 99.67% specificity); SIV – Influenza A Ab Test (95.3% sensitivity, 99.6% specificity) which detects antibodies to nucleoprotein of SIV for serotypes H1N1, H1N2 and H3N2 for swine sera. Manufacturer’s instructions were strictly adhered to during analysis, with positive in-house and also positive and negative test-kit controls incorporated during the serodiagnostic testing.

For each sample, the serostatus was determined for each pathogen of interest using the immunodiagnostic assay. Quantification of the antibody response was determined by colorimetric detection by spectrophotometry using TECAN Sunrise™ microplate reader (Tecan Group Ltd., Männedorf, Switzerland) in conjunction with TECAN Magellan™ data analysis software v7.1 (Tecan Group Ltd., Männedorf, Switzerland). Sample-to-positive (S/P) ratio values for App and Mhyo and sample-to-negative (S/N) ratio values for SIV were extrapolated from the optical density values obtained as per manufacturer’s instructions. Samples with sample-to-positive values ≥0.40 for Mhyo, ≥ 0.50 for APP and samples with sample-to-negative values SIV ≤0.60 were considered as positive as per the criteria given in the manufacturer’s instructions.

### Pluck lesions

At slaughter, lesions of the lungs and heart were visually scored by a single trained observer. The macroscopic enzootic pneumonia (EP) like lesions were scored according to severity using BPEX Pig Health Scheme [28] on a scale from 0 to 55, where 0 indicates no lesion and 55 denotes the extensive presence of the EP-like lesions. Pleurisy was scored using the Slaughterhouse Pleurisy Evaluation System grid, developed by Dottori et al. [29] on a 5 point scale, dependent on lesion location and severity where; 0 = absence of chronic pleuritis lesions; 1 = ventrocranial lesion; 2 = dorsocaudal monolateral focal lesion; 3 = bilateral lesion or extended monolateral lesion (minimum of 1/3 of the diaphragmatic lobe); 4 = severely extended bilateral lesion (minimum of 1/3 of both diaphragmatic lobes). The presence of pericarditis (i.e. purulent inflammation of the pericardium resulting in adhesion of the pericardium to the epicardium and the pericardium with lungs and/or pleura) and heart condemnations were also recorded following the decision of the on-site veterinary inspector.

### Data management and statistical analysis

All data were analysed in R v3.5.2 [30]. Initially, ANOVA tests were performed for sow parity, birth weight and NBA, including data from all 824 animals in the batch that reached slaughter, to check for differences between production flows. Statistical differences were detected for the three variables between each flow. While litter size and NBA were similar between production flows, mean sow parity (2.9 ± 1.50) and mean body weight at birth (1.19 ± 0.30 kg) were lower in flow 3, compared with flow 1 (mean parity = 3.4 ± 1.43 and 1.44 ± 0.28 kg of body weight) and flow 2 (mean parity = 3.3 ± 1.49 and 1.26 ± 0.29 kg of body weight). Additionally, 29% of pigs in flow 3 originated from first parity sows (versus 13.4% of pigs in flow 1 and 19.4% of pigs in flow 2). Therefore, a nested case-control design was applied, whereby pigs originating from each flow were matched by sow parity, birth weight and NBA, resulting in a final data set of 120 in flow 1, 60 pigs in flow 2 and 60 pigs in flow 3. The APP-index (APPI) was calculated for each flow and for the batch of studied pigs. The APPI values are used as a benchmarking tool with regard to the general population. Pleurisy and EP-like lesions were reclassified as present or absent, due to the low number of higher scores recorded. Likewise, due to the low number of sows with parity ≥5, these were re-classified into a single group (i.e. 5+). Due to the low number of negative samples, it was not possible to conduct statistical analyses using qualitative ELISA results and analyses were performed only on ELISA quantitative data.

Residuals of predicted variables were tested for normality using the Shapiro-Wilk test and by examining the normal test plot. Predicted variables were not normally distributed and thus, were analysed using generalised linear mixed models using the *stats* package. A Gamma distribution was fitted to each predicted variable. Univariable linear models were used with S/P or S/N ratio values as predicted variables with early-life indicators (sow parity, NBA, birth body weight, litter size, weaning body weight and cross-fostering status), production flow and pluck lesions (pleurisy, EP-like lesions, pericarditis and heart condemnations) as predictor variables. For all analyses, alpha level for determination of significance and trends was 0.05 and 0.10, respectively. Results for fixed effects are reported as least square means (LSM) ± standard error (SE), and results for continuous variables are reported as the regression coefficient ± SE.

## Results

**Fig. 1:**
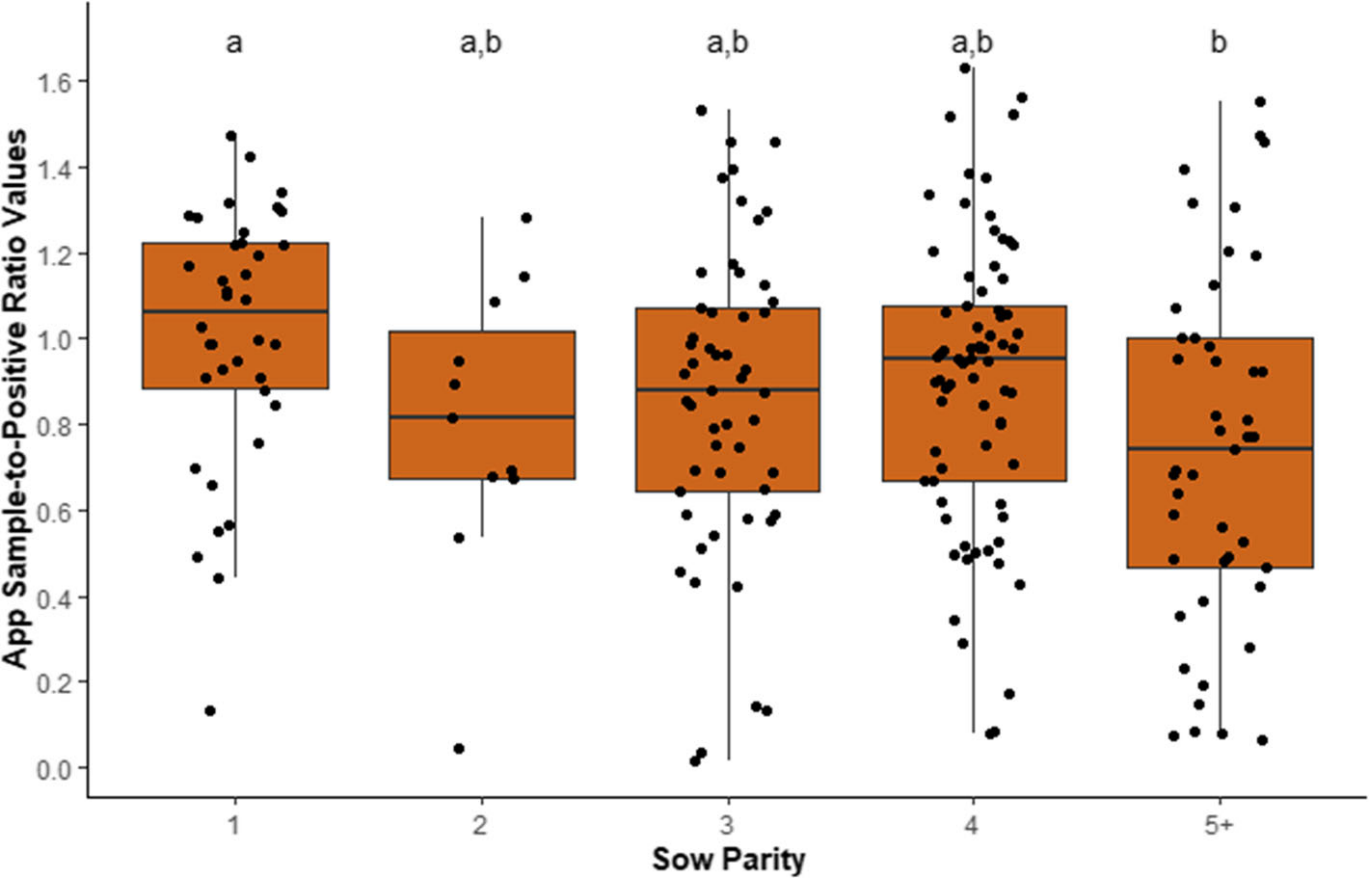
*Actinobacillus pleuropneumoniae* (App) sample-to-positive ratio values by sow parity to App IV toxin for finisher pigs at slaughter (i.e. 24 weeks of age). Pigs were born within 1 week from a single farrowing batch and followed from birth to slaughter on an Irish farrow-to-finish commercial unit. At 24 weeks, regardless of weight, all pigs were slaughtered, and blood samples collected. Sow parity was classified into 5 levels (1, 2, 3, 4 and 5+). ^a,b^ Significant differences between parities; *P* < 0.05

Mean S/P ratio values were 0.87 ± 0.361 (range 0.02 to 1.63) for App, 2.04 ± 0.382 (range 0.53 to 2.8) for Mhyo and mean S/N ratio values were 0.36 ± 0.242 (range 0.06 to 1.09) for SIV. Associations (i.e. *P* values) for the univariable models between the different predictor variables and App and Mhyo S/P and SIV S/N ratio values are presented in Table 1. Results for seroprevalence for App, Mhyo and SIV by sow parity and production flow are presented in Table 2. Pigs born from parity 1 sows had higher App S/P ratio values than those born to parity 5+ sows (1.01 ± 0.069 vs. 0.74 ± 0.046; *P* < 0.05) and there was no difference in App S/P ratio values for pigs born from other sow parity groups (*P* > 0.05; Fig. 1). There was no association detected for any of the three pathogens investigated and other early-life indicators (*P* > 0.05). Seroprevalence for App and SIV decreased as sow parity increased and 100% of samples were Mhyo positive.

**Table 1:** Univariable associations (*P* values) between predictor variables and sample-ratio-values at slaughter for *Actinobacillus pleuropneumonia* (App), *Mycoplasma hyopneumoniae* (Mhyo) and swine influenza virus (SIV) in finisher pigs, born within one week from a single farrowing batch and followed from birth to slaughter on an Irish farrow-to-finish commercial farm

Predictor variables	Value	App	Mhyo	SIV
***Early life predictor variables***				
Sow parity, mean ± SD	3.4 ± 1.33	0.015	0.325	0.718
Number of piglets born alive, mean ± SD	13.2 ± 2.34	0.282	0.393	0.745
Birth weight, kg, mean ± SD	1.2 ± 0.27	0.538	0.976	0.871
Litter size, mean ± SD	14.6 ± 1.99	0.551	0.270	0.919
Weaning body weight, kg, mean ± SD	6.5 ± 1.55	0.797	0.125	0.341
Cross-fostering, %	32.7	0.138	0.806	0.586
***Production flow, n***	223	< 0.001	0.271	0.002
***Pluck lesions***				
Pericarditis^a^, %	16.1	0.441	0.373	0.583
Heart condemnations^a^, %	11.6	0.600	0.119	0.190
Pleurisy^b^, %	25.6	0.489	0.580	0.797
Enzootic pneumonia-like lesions^c^, %	42.1	0.179	0.006	0.740

^a^Scored at slaughter as present or absent

^b^Scored using the Slaugherhouse Pleuritis Evaluation System developed by Dottori et al. [29], from 0 = absence of lesions to 4 = severely extended bilateral lesions and re-classified as present or absent

^c^Enzootic pneumonia-like lesions were ranked according to the BPEX Pig Health Scheme [28] and re-classified as present or absent

**Table 2:** Seroprevalence^a^ by sow parity and production flow^b^ of *Actinobacillus pleuropneumonia* (App), *Mycoplasma hyopneumoniae* (Mhyo) and swine influenza virus (SIV) in finisher pigs, born within one week from a single farrowing batch and followed from birth to slaughter on an Irish farrow-to-finish commercial farm

Predictor variables	App	Mhyo	SIV
***Sow parity***			
1	97.4	100	81.6
2	91.9	100	81.8
3	92.5	100	83.0
4	90.7	100	80.0
5+	76.6	100	70.2
***Production flow***			
1	86.1	100	86.1
2	91.8	100	71.5
3	93.2	100	71.2

^a^Serum samples were analysed using enzyme-linked immunosorbent assay (ELISA) according to the manufacturer’s instructions (IDEXX, Hoofddorp, The Netherlands) for the three respiratory pathogens. Sample-to-positive ratio values were calculated for App and Mhyo while sample-to-negative ratio values were calculated for SIV. Samples with sample-to-positive values ≥0.40 for Mhyo, ≥ 0.50 for APP and samples with sample-to-negative values SIV ≤0.60 were considered as positive as per the criteria given in the manufacturer’s instructions

^b^All pigs were slaughtered at 24 weeks and were retrospectively classified into three production flows according to the time required to move to the next production stage (Flow 1 = normal; Flow 2 = delayed by 1 week; Flow 3 = delayed by more than 1 week). Pigs were selected from each flow in a nested case control study matched by sow parity, birth body weight and number of piglets born alive

**Fig. 2:**
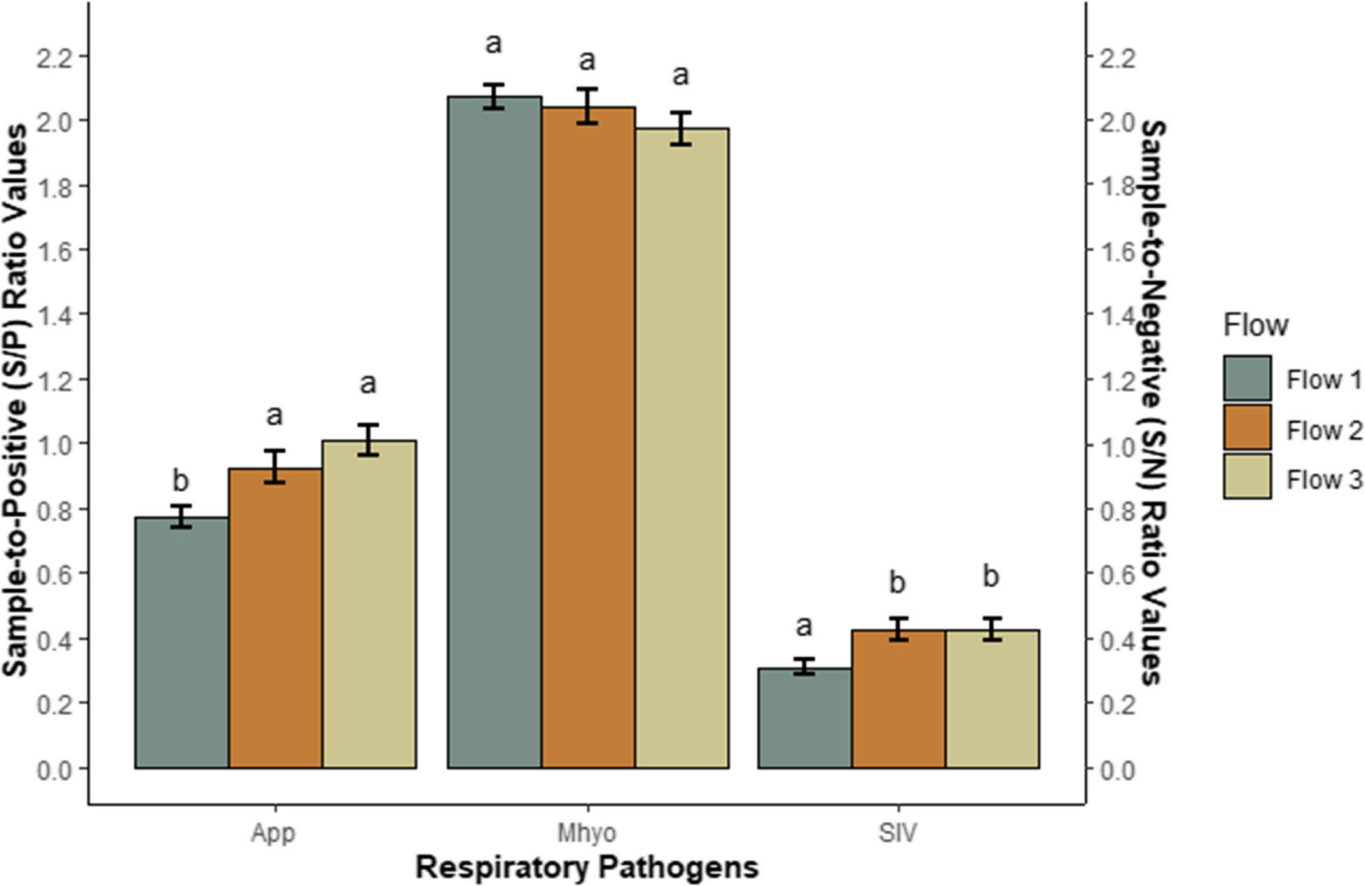
Least square means ± standard error for sample-to-positive (S/P) ratio values for *Actinobacillus pleuropneumonia* (App) and *Mycoplasma hyopneumoniae* (Mhyo) and sample-to-negative (S/N) ratio values swine influenza virus (SIV) by production flow for finisher pigs at slaughter. All pigs were born within 1 week from a single farrowing batch and followed from birth to slaughter on an Irish farrow-to-finish commercial farm. All pigs were slaughtered at 24 weeks and were retrospectively classified into three production flows according to the time required to move to the next production stage (Flow 1 = normal; Flow 2 = delayed by 1 week; Flow 3 = delayed by more than 1 week). Pigs were selected from each flow in a nested case control study matched by sow parity, birth body weight and number of piglets born alive. Swine influenza virus sample-to-negative values are inversely proportional to the quantity of antibodies present.^a,b^ Within each pathogen, significant differences between production flows; *P* < 0.05

Pigs in flow 1 had lower App S/P (*P* < 0.001) and SIV S/N (*P =* 0.002) ratio values compared with pigs in flow 2 and flow 3 (Fig. 2). There were no observed differences in App S/P or SIV S/N ratio values between pigs in flow 2 and pigs in flow 3 (*P* > 0.05). Calculated Mhyo S/P ratio values did not differ between production flows (*P* > 0.05). Seroprevalence for App increased with each subsequent production flow; in contrast, SIV seroprevalence decreased with each subsequent production flow. The overall calculated APPI score for the studied batch of pigs was 0.50 and it increased from 0.28 for flow 1, 0.45 in flow 2 to 0.98 in flow 3.

Pigs presenting EP-like lesions had higher Mhyo S/P ratio values compared with pigs not presenting EP-like lesions (2.1 ± 0.04 vs. 1.9 ± 0.03; *P* = 0.006). There were no differences in App and Mhyo S/P or SIV S/N ratio values between pigs presenting pericarditis, heart condemnations and pleurisy compared with pigs not having such lesions at slaughter (*P* > 0.05).

## Discussion

Previous studies reported an association between early-life indicators and animal health [15, 31, 32]; thus, we hypothesise that these factors could also be associated with antibody response to respiratory pathogens in an endemically infected farrow-to-finish farm. From the six early-life indicators investigated, only sow parity was associated with varying antibody levels in finisher pigs, although this association was only observed with App.

Antibody levels were higher in pigs born from first parity sows compared with sows parity 5 and older, suggesting higher exposure to the pathogen, probably coinciding with a lower passive immunity transferred from gilts to offspring due to inferior quality colostrum [14, 33]. The App ELISA used detected antibodies due to on-farm circulating App infection [34] as the farm did not vaccinate for App.

Contrary to our hypothesis, there were no differences between sow parities in antibody levels for Mhyo or SIV. Although it is difficult to compare with previous studies which report laboratory qualitative results (i.e. positive, suspect or negative) for disease classifications instead of antibody levels, our results are similar to those reported by Sibila et al. [35] and Arsenakis et al. [36] who did not find an association between sow parity and *Mycoplasma* colonisation in piglets during the lactation period. However, reports in the scientific literature regarding the associations between sow parity and transmission of Mhyo to their progeny are contradictory. For example, Fano et al. [13] reported that offspring from lower parity sows were more likely to be infected by Mhyo and Calsamiglia and Pijoan [37] reported an inverse relationship between sow parity and the likelihood of Mhyo transmission, suggesting that predictability of piglet Mhyo status based on sow parity remains unclear. A limitation of our study is that sow antibody levels were not determined, and therefore the variability of antibody levels for the pathogens of interest regardless of sow parities present in the farm is unknown. Nonetheless, the lack of associations between sow parity and antibody levels for Mhyo and SIV observed in the present study could be related to the farm vaccination protocol. Like many commercial pig units, the studied farm immunised the pigs against Mhyo and SIV. It is likely the administered Mhyo vaccination directly boosted antibody levels in susceptible piglets and the blanket vaccination of sows against SIV supported the developing immune system of the piglet due to the transfer of maternally derived antibodies via colostrum [38], consequently advancing herd immunity. Indeed, elevated antibody levels for Mhyo and SIV were recorded in piglets regardless of their dam parity. The absence of associations between antibody levels for App, Mhyo or SIV and other early-life indicators could be partly explained by the cross-fostering practices applied in the farm where the study was conducted. A substantial proportion (33%) of pigs were cross-fostered. Pigs were cross-fostered after 12 h post-farrowing with exceptions for pigs from larger litters being cross-fostered earlier to match sows’ rearing capacity with litter size, and pigs with lower birth body weight cross-fostered later during lactation enabling maximal colostrum intake from their dam (for more details please refer to Calderón Díaz et al. [39]).

Production flow was associated with antibody levels for App and SIV, confirming the relationship between management practices and pig health. Pigs in flow 2 and flow 3 shared air space (i.e. flow 2) or were re-mixed (i.e. flow 3) with pigs that had returned from the hospital facilities having recovered from illness and/or injury [27]. During this post-weaning period, passive immunity from the sow is in decline while the piglet’s active immune system is not yet fully developed. The resulting immunity gap coincides with the stressful timing of weaning and disease pressure introduced by new pen-mates. This facilitated re-circulation of disease and a higher and earlier risk of exposure to pathogens [34] compared with pigs in flow 1, due to contact between animals of different ages and mixed immune status [40]. *Actinobacillus pleuropneumonia* circulates in the herd for long periods of time [5], with antibody levels increasing with age [41]. Therefore, the higher antibody levels for App from pigs in flow 2 and flow 3 could be associated with direct and prolonged exposure to recovered sick pigs [27] that could still be carrying and shedding pathogens [42]. Similarly, the calculated APPI scores were higher in flow 2 and peaked in flow 3 than in flow 1 indicating that delaying pigs from moving in a timely manner through the different production stages is negatively associated with pig health. However, whether this association is causative or explanatory warrants further investigation. On the contrary, as SIV is episodic [8], the lower antibody response observed from pigs in flow 2 and flow 3 infers an earlier exposure time-point to this pathogen compared to pigs in flow 1, as the antibody levels detected have rapidly declined post-infection [43]. This highlights the importance of implementing a strict AIAO policy in pig farms to minimise disease exposure, especially during periods of increased infection pressure. However, as this is difficult to do in farrow-to-finish farms, an ‘all-forward’ policy might be more easily adhered to, whereby no pig is left behind from stage to stage but rather they are split marketed at the point of slaughter. Alternatively, special attention to slow growing pigs during the grow-finisher period and to previously hospitalised pigs should be implemented by relocating these animals away from the main facility, ensuring strict implementation of the AIAO system. Instead, the higher antibody response for SIV observed from pigs in flow 1 indicates on-farm viral re-occurrence [43]. Finally, the similar Mhyo S/P ratio values between production flows could be partly due to the aforementioned Mhyo vaccination protocol but such elevated response at slaughter is probably due to natural infection [38], or reinfection with a different strain of Mhyo throughout the unit [44].

Although the result of higher antibody levels for Mhyo in pigs with presence of EP-lesions was expected, there was a high percentage of pigs (42.1%) presenting lesions despite the farm vaccinating piglets against Mhyo. However, vaccinating for Mhyo is reported to reduce clinical signs, and decrease infection levels [35, 45]; and thus, the percentage of EP-like lesions could be higher if this farm was not vaccinating [45, 46]. Vaccines may be ineffective for various reasons including vaccine administered at the incorrect age of the pig, non-adherence to vaccine schedule, insufficient time between vaccination and exposure to the disease, pig infected at the time of vaccination either by the pathogen of interest or a concurrent infection and a vaccine not being efficacious towards strains circulating on the farm. Strains of Mhyo differ in virulence [47] with a variety of commercial bacterin vaccines available [48]. Villarreal et al. [47] suggested that vaccination with a low virulent Mhyo isolate does not provide cross-protection against a more virulent counterpart [47].

We hypothesised that mean antibody levels would be higher for pigs presenting pluck lesions at slaughter; this was only observed between EP-like lesions and Mhyo antibody levels. The lack of the expected association between other pluck lesions and antibody levels in this study may be explained by the amount of time required for the development of a macroscopic lung lesion, resulting in the absence of the lesions at slaughter. Other possibilities include the ability of the lesion to resolve, or to the fact that pluck lesions are multifactorial and multiple pathogens can cause them [28].

Finally, when interpreting the results, it is worth noting/considering that results represent finding from only one farrow-to-finish farm and a small sample size. Management and vaccination practices, sow parity distribution and on-farm prevalence of respiratory pathogen vary between farms and thus, results would likely differ if we were to include more farms and/or sample more pigs. However, the merit of this study is the fact that there is scarce information available in the scientific literature about animal and herd management factors, serological response to respiratory pathogens and pluck lesions at an individual animal level; most studies report such relationships on a farm level. Also, it seems that although a high proportion of Irish farmers claimed to practice strict AIAO during the grower-finisher period, they mix older pigs younger pigs in the different production stages [27]. Also, as previously observed by Calderón Díaz et al. [26], delaying pigs from the normal production flow seems to be a common practice in Irish farms, and possibly in other countries, to avoid financial penalties imposed by the abattoir if pigs fall outside the established body weight range [49]. It is not possible to discern if associations reported in this study are causative or exploratory and thus, controlled studies should be carried out to investigate this further.

## Conclusion

Sow parity and production flow were associated with variation in antibody levels for the investigated respiratory pathogens. Offspring from first parity sows had more elevated App antibody levels, indicating the importance of adequate gilt management programs due to the high seropositivity detected. The inclusion of an App vaccine would assist with improved passive immunity and increased immune development of their progeny. The differences observed between the production flows in the detected antibody levels for App and SIV reflect moving pigs in a timely manner is beneficial to reduce on-farm disease circulation. Enforcement of a meticulous AIAO production system would minimise adverse effects of respiratory disease on pig performance and health. Under the conditions of this study, only an association between Mhyo and presence of EP-like lesions was observed. Furthermore, although the farm vaccinated piglets against Mhyo, extension of the programme to include the sows and gilts may be beneficial to controlling the infection spread within the herd.

## Data Availability

The datasets used for the results presented in this study are available from the corresponding author upon reasonable request.
